# Modification of a pilot-scale continuous flow reactor for hydrothermal liquefaction of wet biomass

**DOI:** 10.1016/j.mex.2019.11.019

**Published:** 2019-11-19

**Authors:** Feng Cheng, Travis Le Doux, Brian Treftz, Scott Woolf, Jiuling Yu, Juanita Miller, Umakanta Jena, Catherine E. Brewer

**Affiliations:** Department of Chemical and Materials Engineering, New Mexico State University, P.O. Box 30001 MSC 3805, Las Cruces, NM 88003, USA

**Keywords:** Pilot-Scale Continuous Flow Hydrothermal Liquefaction of Biomass, Hydrothermal liquefaction, Continuous flow reactor, High-pressure filtration, Microalgae

## Abstract

A pilot-scale continuous flow reactor (CFR) was modified for hydrothermal liquefaction (HTL) of algae slurry under subcritical conditions to investigate the feasibility of scaling up from batch to continuous processing. Modifications included a novel dual filter system that can remove solids at system pressure and temperature, and undergo in-situ cleaning. Commissioning was carried out to address potential particle settling and clogging problems, and to estimate reactor transport characteristics. CFR performance was evaluated by running 31.4 L algae slurry with solids loadings of 3−5 wt.% under 325−350 °C and 18 MPa for 7 h. C and N elemental yields in HTL aqueous phase reached 39.0 wt.% and 61.8 wt.%, respectively. Future improvements to the CFR system will focus on higher solids loading and addition of in-line HTL liquid upgrading capabilities following the filtration system.

• A high-temperature, high-pressure filtration system was designed to remove solids from HTL liquid/gaseous products at near reaction conditions to keep heavy oils in the liquid phase.
• Uninterrupted reactor operation was achieved by cycling between the dual filter systems and performing in-situ filter cleaning.
• Measured reactor residence time distributions were narrow and close to the calculated theoretical mean time.

## Graphical abstract

**Figure:**
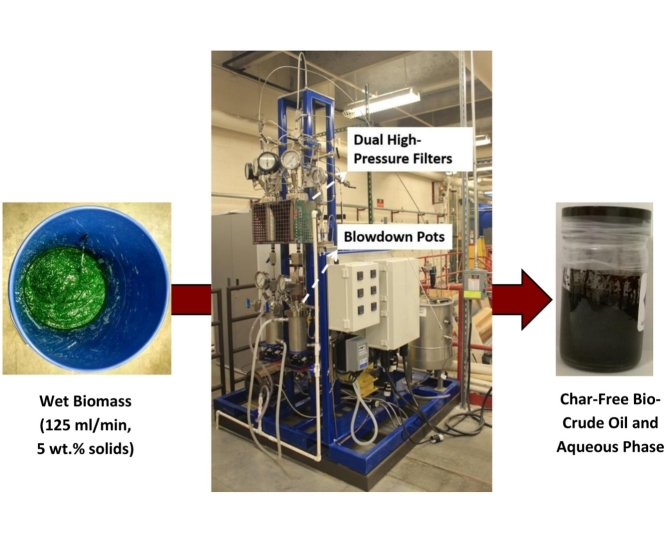

**Specification Table**

**Table:** 

Subject Area:	Chemical Engineering
More specific subject area:	Hydrothermal Conversion of Wet Biomass into Biofuel
Method name:	Pilot-Scale Continuous Flow Hydrothermal Liquefaction of Biomass
Name and reference of original method:	Mørup, Anders Juul, et al. "Construction and commissioning of a continuous reactor for hydrothermal liquefaction." Industrial & Engineering Chemistry Research 54.22 (2015): 5935–5947.
Resource availability:	https://pubs.acs.org/doi/pdf/10.1021/acs.iecr.5b00683?rand=ldfthbvp

## Method details

### Background

Hydrothermal liquefaction (HTL) is an energetically favorable thermochemical conversion technology for wet biomass [1] because HTL does not require feedstock drying [2] and HTL is able to convert most biomass components into bio-crude oil [3]. HTL reactions are catalyzed by H^+^ or OH^−^ ions [[2], [3], [4]] released from water molecules under subcritical conditions (180−370 °C and 5−21 MPa). When approaching its critical point, water possesses both liquid- and gas-like properties, with lower density and increased mass transfer abilities. The lower dielectric constant of water at high temperature is conducive to dissolving more organic molecules derived from biomass and facilitating HTL reactions. HTL involves hydrolysis, dehydration, decarboxylation, repolymerization, and deamination [2,5], breaking biomolecules such as lipids (e.g. ω-3 fatty acids), proteins, and carbohydrates [6,7] into smaller organics to form bio-crude oil, aqueous, char, and gaseous phases. Like other thermochemical conversion processes, the yields and characteristics of the product phases depend on the reaction conditions, with higher heating rates and shorter reaction times favoring bio-crude oil products [8]. Such reaction conditions are easier to achieve in a continuous-flow, steady-state reactor systems since one does not have to heat up and cool down the entire thermal mass simultaneously. Continuous flow systems, however, have to address the challenges of slurry flow and four-phase product separation under high temperatures and pressures.

#### Objectives of this study

In this study, a pilot-scale continuous plug flow reactor was fabricated to achieve in-situ char filtration under full HTL temperature and pressure without interrupting system operations. This was possible through a novel double cylinder filtration system with blow-down pots and a high-pressure gas booster. The focus of the work was on fluid flow and product separation, with future work to focus on heat integration and energy efficiency. The final goal of this project is to improve the capability of handling and processing biomass slurries in CFR systems to make progress towards energy recovery from abundant, low-cost wet biomass waste streams such as microalgae grown on municipal wastewater.

#### Slurry flow mechanisms in continuous flow reactors

Management of slurry flow to prevent clogging requires understanding of the flow mechanisms, including fluid flow patterns, terminal velocity, and residence time distributions in a pipe/reacting system. Here, the algae biomass slurry was characterized by small organic particles (2−80 μm) in subcritical water, slow flow velocity (<1 m/s), and the horizontal and vertical tubing with inner diameters (I.D.) ranging from 1 mm to 8 mm (immediately after the reactor, before the filters). These conditions were different than those that are generally found in studies on slurry flow: large inorganic particles (> 0.1 mm [9]), high flow rates (>1.5 m/s [10]), horizontal tubing with diameters of 2–5.5 cm [11], and atmospheric pressure conditions. Therefore, evaluation of the general flow conditions was needed to guide reactor design.

Velocity profiles of slurry flow in pipes are influenced by particle density [12], particle size [13], particle velocity, solids concentration [14], and distance from the tube wall. At the region near the tube wall, a higher velocity contributes to the intact “streaky” structure of flow in single-phase flow. By comparison, low velocity deteriorates the typical streaky flow structure at the region near the wall, and a thick and well-packed bed is formed—the start of clog formation [15]. The velocity profile of the solids in the tube is characterized by higher velocity in the upper half of the tube relative to the lower half, with a coarser, particle-rich sliding bed regime in the lower part of the tube due to gravity [16]. The smaller the particle sizes and the lower the solids concentration, the more symmetrical the velocity profiles are as gravity and viscous shear stress have less influence [12]. The asymmetry of the velocity profile decreases at higher flow rates [17]; lower flow rates decrease turbulence and eddying, causing solid sedimentation. In this study, smaller diameter tubing was chosen to achieve as high of velocity and as symmetrical a flow as possible.

Tube shape also impacts the velocity profile and, therefore, the tendency towards sedimentation. At a tube bend, the asymmetry of the velocity profile is lower downstream of the bend than in a straight horizontal tube, owing to the strong turbulence effect of fluid at the bending point [18]. Zhang et al. [19] found that most erosion occurs about 40° into an elbow due to the direct hit from particles flowing through the tube. This abrasion point moves deeper into the elbow as the flow velocity increases or the bend orientation changes due to gravity force. Clogging is more prevalent in bends with steeper angles. Later parts of the bend experience less abrasive force to keep particles moving. A similar change of velocity profile is also observed at the tee of a horizontal tube with a vertical branch (a higher solids concentration was found in the blank zone of the tee-junction) and in a U-shaped bend [20]. In this study, therefore, all bends were kept as smooth as possible and 90° elbows were avoided.

The plug flow reactor portion of the system was vertically oriented, which would suggest an interaction of gravity with fluid flow through the reactor. Few studies have been conducted for slurry flow in a vertical tube. Sumner et al. [21] found that if the solids concentration is lower than 40 vol.%, and the particles are smaller than 500 μm, the profiles of solid concentration are basically consistent throughout the entire tube. In this study, with solids concentrations of <10 wt.%, and particles between 2−80 μm, influence of flow characteristics in the vertical tube was assumed to be negligible relative to that in a horizontal tube.

Pressure drop is an important parameter for consistency of the flow pattern and for selection of pumps. According to the Darcy-Weisbach equation of pressure drop (Eq. (A1)) [22], larger pipe diameters (the larger cross-section area) lead to lower pressure drop in the pipeline, and lower energy consumption for the feed pump.

(A1) $$\frac{\Delta P}{L}=\frac{8\rho Q^{2}f}{\pi^{2}D^{5}}$$

where *ρ* is the density of the fluid in kg/m^3^; *Q* is the volumetric flow rate in m^3^/s; *f* is the Darcy friction factor; *L* is the length of the pipe in m; and *D* is the hydraulic diameter of the pipe in m. Pressure drop is impacted by flow velocity [23], solids concentration [12], and particle size [24]. Lower flow rates decrease the pressure drop until the flow velocity is lower than the critical deposition velocity [25], at which particle settling can occur. Particle settling in the slurry leads to higher pressure drops due to creation of smaller cross-sectional area and more friction losses. Pressure drop is rarely affected by particle size at low solids concentrations since the contribution of particle texture to fluid friction is negligible [26]. At higher flow velocities, pressure drop is more sensitive to solids concentration. At lower flow rates and higher solids concentrations, pressure drop is influenced more by fine particles than course particles. Anticipating the small particle size of the HTL solids in this study, larger diameter tubing (increased by 20–120 %) was used between the main reactor and the filter, where the pressure drop was higher, compared to the rest of CFR system.

An essential parameter for determining a safe operational window of flow rate is the terminal velocity, below which the particles in the flow would start to deposit. Since the terminal velocity of flow in tubing can be measured according to Newton’s law and is correlated to the density and size of particle, fluid density, and drag coefficient, the operational flow rate should be tuned based on various operating conditions, and properties of particles and fluid.

#### Residence time distribution

The residence time distribution (RTD) is critical to estimate the probability distribution of time that a reactant stays in the reactor. Measurement of RTD is based on comparison of the concentration of a tracer to the theoretical space time, τ (in minutes):

(A2) $$\tau =\frac{V}{F}$$

where *V* is the inner volume of the reactor in mL, and *F* is the volumetric flow rate in mL/min. In continuous flow reactor systems, especially reactors in which fluid temperature and pressure change substantially, estimations of RTD are more complicated than the uniform residence time in batch reactors. Evaluation of the RTD function involves normalizing the function, E(Θ), and plotting against the relative time, Θ [27,28]. To measure the RTD in a subcritical water system, Kruse et al. [28] suggested 1) injecting tracers without heating, 2) equipping the view cell with a powerful heating system, 3) using stable aromatic compounds as tracers, and 4) detecting concentration of the aromatic tracer by UV-Vis spectrometry. The RTD function is then extracted from the concentration and time data using a convolution integral, either in the time domain or the frequency domain. Convolution in the time domain was preferable for RTD extraction due to higher data dependability [29]. In the time domain analysis, the flow model was assumed by fixing the shape of the RTD curve, and including the mean residence time, *τ,* and the dispersion, *σ*^2^. To obtain more reliable RTD data from a continuous system by time domain analysis, the Plug Flow with Dispersion model at large values was adopted [30]. In this study, the real residence time was simply defined as the RTD of the flow in the entire CFR system at a desired reaction temperature, regardless of a non-uniform temperature profile throughout the continuous system.

### Configuration of the CFR system

**Fig. 1:**
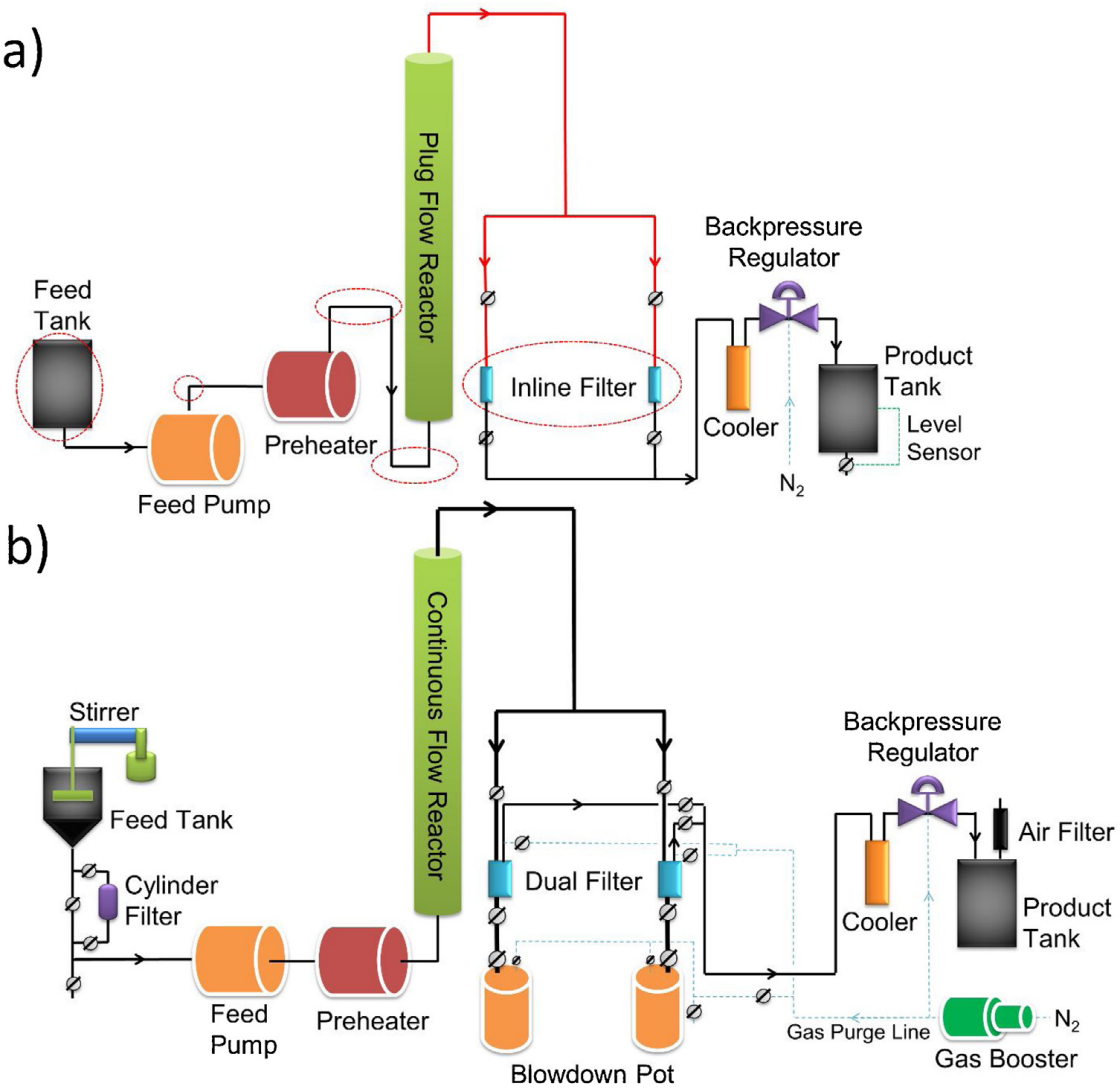
The pilot-scale CFR system before (a) and after (b) modification.

Schematics of the original and modified pilot-scale continuous flow reactor (CFR) system for high-pressure, high-temperature hydrothermal liquefaction are shown in Fig. 1. The original CFR system consisted of a feed tank, high-pressure metering pump, coil preheater, vertical plug-flow reactor, dual inline filters, cooling coil heat exchanger, back-pressure regulator, and product tank. The flat-bottom feed tank, sharp angles of tubing bends, undersized tubing for transferring the HTL solids-containing stream, undersized inline filter, and low-pressure nitrogen supply system (10.3 MPa) had to be modified to enable conversion of high solids loading feedstock into bio-crude oil safely and efficiently (Fig. 1a). In the modified CFR system, a dual high-pressure filter system with blow-down vessels for char collection, and a high-pressure gas booster were introduced, as the core components to solve the clogging and pressure problems.

The modified CFR system has five component systems: (1) the feed supply system, (2) the reaction system, (3) the separation system, (4) the pressure letdown system, and (5) the auxiliary systems. As shown in Fig. 1b), algae slurry enters from a stirred tank, through a cylinder filter, and is pressurized in a metering pump. The slurry is then heated by a preheater prior to entering the vertical plug-flow reactor from the bottom. After exiting from the top of the reactor, the HTL products enter one of the high-pressure filters where solid residues are removed by the filter elements and collected periodically in the blow-down pots underneath the filter vessels. The solids-free bio-crude oil/aqueous phase/gas phase is then cooled in a water-chilled condenser and the pressure reduced to ambient conditions through a back-pressure regulator. Finally, gaseous products are vented from the liquid products in a flat-bottom product drum. Materials of construction and specifications of the components used in construction of the CFR system are provided in the supplemental information (Table S1).

#### Supply system

The supply system consists of a cylindrical feed tank (114 L) with a C-clamp mounted agitator, a cylinder filter (pore size = 841 μm), and a metering pump (Fig. S1). The cylinder filter is used to remove larger particles (e.g. rocks, soil, biological debris) from the slurry to prevent blocking thinner tubing downstream. The filter is equipped with a pass-by valve that can be opened to clean the filter without disturbing the feedstock supply. The metering pump provides pressures up to 34.5 MPa with flow rates between 0−250 mL/min. A pulsation dampener, set at approximately 80 % of the system pressure, on the metering pump stabilizes the feed flow. A pressure transducer provides continuous system pressure monitoring.

#### Reaction system

The reaction system consists of a preheater and a vertical plug-flow reactor (PFR). A mild temperature of 133 °C [31] is recommended for the preheater to prevent liquefaction reactions from occurring before the PFR. The instant heating rate is designed to be 150 °C/min in the 4.5 kW preheater using a short tube coil. A PFR configuration was selected because the higher achievable heating rates [32]. According to the estimated physical properties of the biomass slurry (Table S2), three ceramic heaters are used to supply heat for the PFR: 4 kW for the lower heater and 2 kW for the middle and upper heaters, with layers of fiberglass insulation providing insulation. The material details of the preheater and PFR are shown in Table S1.

#### Separation system

A novel component of this CFR is the set of parallel high-pressure cylinder filters connected to blow-down pots designed to effectively remove solids (e.g. ash and char) from the hot, pressurized HTL product flow. In selecting the placement of the filters, three options were considered: (1) before the cooler (high temperature, high pressure), (2) between the cooler and the back-pressure regulator (ambient temperature, high pressure), or (3) after the back-pressure regulator (ambient temperature and pressure). Previous batch experiments had shown that separation of HTL solids is difficult once the char/oil mixture has cooled because heavy bio-crude oils form a thick layer of asphalt-like filter cake with the char particles [33]. Therefore, the filter was placed before the cooler. HTL products enter the dual filter system at approximately 220 °C and system pressure (∼17.3 MPa). Under such conditions, the oily components are less viscous and more evenly dispersed in the liquid phase, enabling char capture while the bio-crude oil passes through the filter. Each filter housing volume is 1.9 L. The filter has a pore size of 10 μm and an area of 405 cm^2^. More severe reaction conditions (higher temperature and higher pressure) are beneficial for achieving better separation efficiency, while still staying below the rated temperature and pressure of the filter vessels (Table S1). The relatively large surface area of the filter, and the low velocity (130−180 mL/min) of the entering stream, increase the time that the filter can operate before cleaning (>4 h for HTL of 5 wt.% algae slurry). A diaphragm-type differential pressure (DP) gauge is used to monitor pressure outside and inside the filter element. Filter cleaning is initiated as soon as the differential pressure reaches 0.34 MPa.

**Fig. 2:**
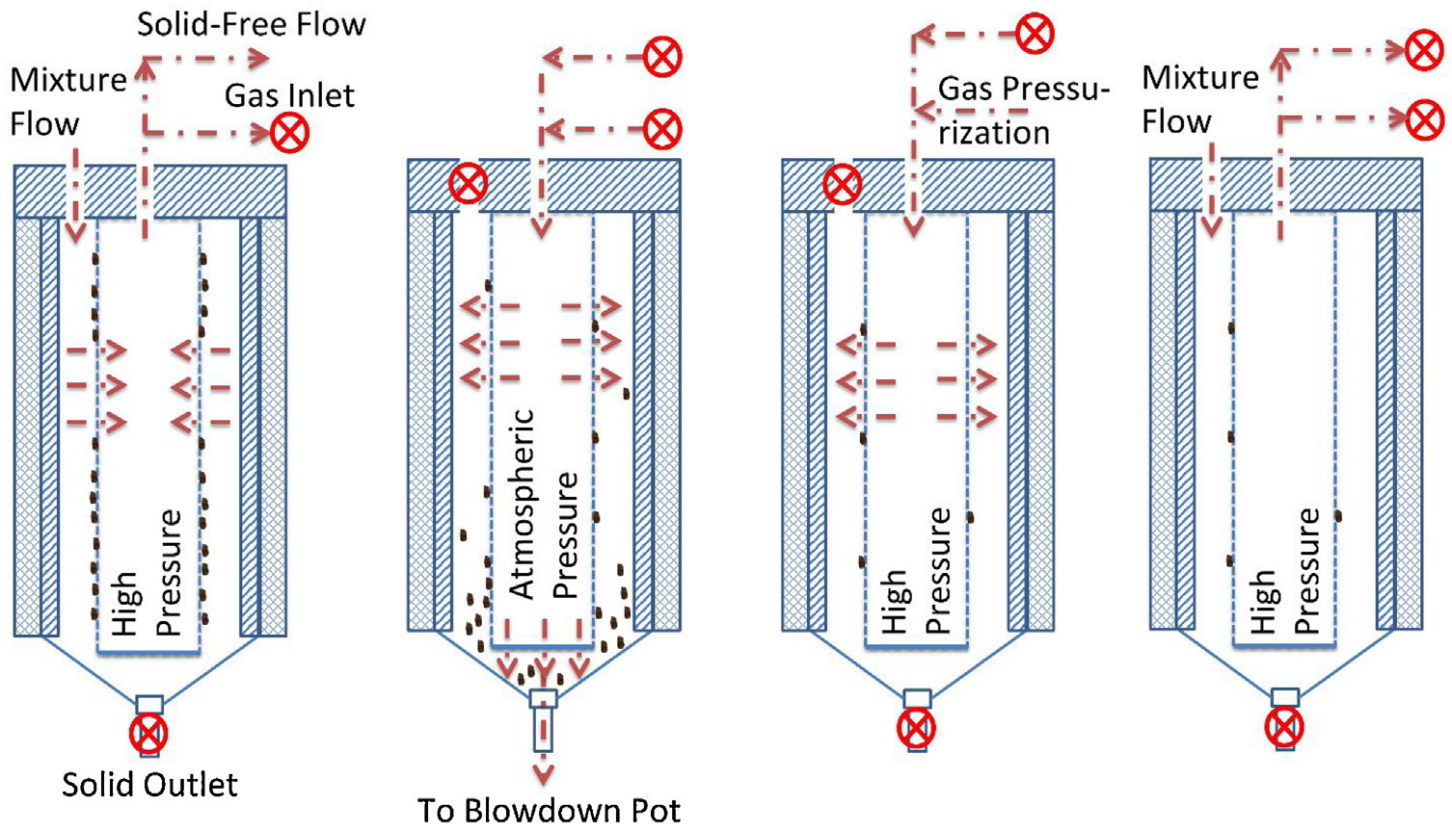
Filtration and in-situ cleaning processes of the dual filter system.

The in-situ filter element cleaning process is diagrammed in Fig. 2. The filter vessel has four valves: feed inlet, product outlet, purge gas inlet, and solids outlet. During regular operation, the solids outlet and the purge gas inlet are closed. Solids-containing feed enters the outer portion of the filter housing; liquids and gases cross the filter element and exit from the top center product outlet. For cleaning, both inlets and outlets of the running filter vessel are closed and HTL product flow is guided to the other filter vessel. Solids are removed from the filter vessel by manually opening the solids outlet valve to the ambient-pressure bow-down pot. The downward force on the solids from the pressure difference “scrapes” the solids off of the vertically-aligned filter element surface and into the blow-down pot through a short, wide (7.9 mm I.D.) connector. Water-jackets on the blow-down pots (Fig. S2) cool the solids to ambient temperature, after which the pot is vented with a relief valve and the solids transferred to a storage drum through a screen (25 μm). Once the filter vessel has been emptied, the solids outlet is closed and the filter vessel pressurized back to system pressure with compressed nitrogen using the gas booster. Re-pressurization and gradual valve opening help prevent violent water evaporation when the cleaned filter vessel is reconnected to the feed inlet. At the system flow rates, refilling of a cleaned filter vessel with fresh HTL product takes approximately 20−30 min, during which the main outflow of the CFR stops even though the reaction continues uninterrupted. The filter vessel refilling time did not appear to affect the HTL liquid productivity over a 7-h reactor run time.

#### Pressure letdown system

Solids-free liquid and gas products from the filter pass through a heat exchanger containing a water-cooled coil (Fig. S3), then through a back-pressure regulator (BPR) into the product collection vessel. At the cooler outlet, a thermocouple is used to monitor flow temperature and prevent hot fluids (>70 °C) from entering the BPR and damaging the diaphragm. Nitrogen gas from a pressurized cylinder and an air-driven gas booster provides pressure to the dome-side of the BPR diaphragm. After cooling and depressurization, liquid and gaseous HTL products enter an ambient-pressure metal drum (69 L) where the gaseous products vaporize. The drum’s vent is equipped with an activated carbon air filter (details in Table S1) to manage odors (which proved to be a major consideration as the distinct and unpleasant smell of “cooked algae” can be detected in other rooms (nearby offices) in the building).

#### Auxiliary systems

A set of check valves and rupture discs (rated for 31.4 MPa at 350 °C) was installed between the pressure transducer and the preheater to protect all upstream instruments from reverse flow of high-temperature slurry. An auto relief valve (rated at 20 MPa) and a manual relief valve were installed after the PFR to enable venting in the case of clogging. Another rupture disc (rated at 31.4 MPa at 350 °C) was located after the PFR. Rupture discs (19.2 MPa at 350 °C) on each of the filter vessel lid assemblies are used to protect the downstream components in the case of rapid filter clogging. The outlets of all rupture discs were plumbed (with gentle curving shapes) to a stainless-steel tank to direct high-pressure, hot slurry flow to a safe collection point in the case of over-pressurization. During commissioning, one clog did result in the rupturing of one disc, the consequences of which were limited to a loud noise, the replacement of the rupture disc, and the cleaning of the outlet tubing and collection tank—such precautions are highly recommended for anyone designing these kinds of systems to prevent injury and damage.

Instrumentation on the CFR included six thermocouples: between the preheater and the PFR, within the lower, middle, and upper zones of the PFR, within the filter vessels, and between the cooler and the BPR. The thermocouples in the preheater/PFR were connected with micro-controllers and solid-state relays for programming of the band heaters. In addition to the differential pressure gauges on the filter vessels, seven regular pressure gauges were used on the CFR: at the outlet of the metering pump, at the outlet of the PFR, on each of the two filter vessels, on each of the two blow-down pots, and at the inlet of the cooler.

#### Reactor maintenance

Between reaction runs, maintenance is needed to remove organic and inorganic residues from the CFR system, because: (1) solid residues decrease tubing cross-sectional area, increasing the pressure drop and likelihood of clogs; (2) solid particles near the settled bed periphery with low flow velocities cause overheating due to the screen effect [15]; (3) product residues contaminate future samples, and (4) fouling leads to lower heat transfer efficiency. Organic solid residues can be removed by pumping 8 L of 12.5 wt.% alkaline degreaser (KYZEN, Nashville, TN, USA) through the CFR system, followed by a rinse with clean water at 300−350 °C and 10–17.3 MPa. After the rinse, the temperature is decreased to 125 °C and the pressure in the BPR is released rapidly, leading to formation of turbulence to loosen sediments in dead zones. A similar cleaning method was used by Mørup et al. [34]. The stainless-steel filter elements are cleaned by combustion in a muffle furnace at 545 °C for 6 h to remove organics, and soaking in 2.7 L of 1 M nitric acid overnight to remove ash. The PTFE diaphragm, the delicate part in the BPR, needs to be cleaned periodically with soap and water to eliminate oily residues.

## CFR commissioning

Commissioning of reactor occurred in three phases: water flow at progressively higher temperatures and pressures, characterization of the reactor residence time distribution, and conversion of microalgae at different solids loadings to confirm continuous operation. The continuous operation target was 5 wt.% solids loading, 350 °C reaction temperature, and uninterrupted processing of at least 30 L of algae slurry (approximately 7 h of operation).

### Estimation of residence time distribution

The residence time distribution (RTD) in the CFR system was estimated using a method modified from [34]. A steady flow of water through the CFR was established at the desired temperature (25 °C and 350 °C), pressure, and flow rate conditions. A volume (1.5 L) of phenol tracer solution (60 mg/L) was injected into the feed tank and samples of reactor effluent were collected after the BPR. The concentration of phenol in the effluent was measured by a Lambda 35 UV/Vis spectrometer (PerkinElmer, Inc., Waltham, MA, USA). Parameters about the flow RTD were calculated from the concentration vs. time data based on the following equations [35]:

(1) $$E\left(t\right)=\frac{c(t)}{\int_{0}^{\infty }c(t)dt}=\frac{c_{i}}{\sum c_{i}\Delta t_{i}}$$

(2) $$t_{m}=\int_{0}^{\infty }tE(t)dt=\frac{\int_{0}^{\infty }tc(t)dt}{\int_{0}^{\infty }c(t)dt}=\frac{\sum t_{i}c_{i}\Delta t_{i}}{\sum c_{i}\Delta t_{i}}$$

(3) $$\sigma^{2}=\int_{0}^{\infty }{\left(t-t_{m}\right)}^{2}E(t)dt=\frac{\sum {\left(t_{i}-t_{m}\right)}^{2}c_{i}\Delta t_{i}}{\sum c_{i}\Delta t_{i}}$$

(4) $$\frac{\sigma^{2}}{t_{m}^{2}}=\frac{2}{Pe}+\frac{8}{{Pe}^{2}}$$

(5) $$E\left(\theta \right)=\frac{1}{2\sqrt{\frac{\pi \theta }{Pe}}}\text{e}\text{x}\text{p}\left[\frac{-{\left(1-\theta \right)}^{2}}{\frac{4\theta }{Pe}}\right]$$

where *c*(*t*) is the instant concentration at the outlet, *E*(*t*) is the exit age distribution, *t_m_* is the mean residence time, *σ* is the time variance, *θ* is relative time (*t* / *t_m_*), and *Pe* is the Peclet number. The value of *Pe* was obtained experimentally by determining *t_m_*, and *σ*^2^ from the RTD data and solving Eq. (4) for *Pe*. *E*(*θ*) is the normalized distribution function which shows how the added tracer exits the reactor relative to the expected residence time.

### Conversion of wastewater treatment algae

HTL experiments consisted of processing 31 L of algae slurry, with solids loadings of 3 and 5 wt.% (confirmed by freeze drying feedstock samples), at 325−350 °C, theoretical retention times of 3−9 min (obtained by dividing the total volume of PFR by the instant flow rate), and flow rates of 152−155 mL/min. The temperature and pressure profiles in the PFR and throughout the entire CFR system were monitored at regular intervals. Liquid products were collected in 25 mL aliquots every 10−20 min, and stored at 4 °C before analysis. Solid products were collected from the blow-down pots, freeze-dried, and stored in a desiccator prior to characterization. Yields of carbon and nitrogen in HTL liquid product were estimated using:

(6) $$\text{Y}\text{i}\text{e}\text{l}\text{d}=\frac{C_{\text{H}\text{T}\text{L}\;\text{l}\text{i}\text{u}\text{q}\text{i}\text{d}}\;\times \;F_{\text{H}\text{T}\text{L}\;\text{l}\text{i}\text{q}\text{u}\text{i}\text{d}}}{C_{\text{d}\text{r}\text{y}\;\text{a}\text{l}\text{g}\text{a}\text{e}}\;\times \;S_{\text{d}\text{r}\text{y}\;\text{a}\text{l}\text{g}\text{a}\text{e}}\;\times \;F_{\text{a}\text{l}\text{g}\text{a}\text{e}}\;}\times 100\text{\%}$$

where *C*_HTL liquid_ is the elemental concentration in HTL liquid in g/mL; *F*_HTL liquid_ is the volumetric flow rate in mL/min; *C*_dry algae_ is the elemental composition in dry algae in wt.%; *S*_dry algae_ is the solids loading of dry algal in the feed flow in wt.%; and *F*_algae_ is the mass flow rate of algae slurry in g/min.

A modified nitrogen to carbon (N/C) ratio in HTL liquid product was calculated using:

(7) $$\text{N}/\text{C}\;\text{r}\text{a}\text{t}\text{i}\text{o}=\frac{\text{T}\text{o}\text{t}\text{a}\text{l}\;\text{n}\text{i}\text{t}\text{r}\text{o}\text{g}\text{e}\text{n}\;\text{i}\text{n}\;\text{H}\text{T}\text{L}\;\text{l}\text{i}\text{q}\text{u}\text{i}\text{d}\;\text{p}\text{r}\text{o}\text{d}\text{u}\text{c}\text{t}}{\text{T}\text{o}\text{t}\text{a}\text{l}\;\text{o}\text{r}\text{g}\text{a}\text{n}\text{i}\text{c}\;\text{c}\text{a}\text{r}\text{b}\text{o}\text{n}\;\text{i}\text{n}\;\text{H}\text{T}\text{L}\;\text{l}\text{i}\text{q}\text{u}\text{i}\text{d}\;\text{p}\text{r}\text{o}\text{d}\text{u}\text{c}\text{t}\;}$$

### Characterization of HTL liquid product

Total organic carbon (TOC) and total nitrogen (TN) of aqueous phase were measured using a model TOC-V_CPH_ analyzer (Shimadzu Corp., Kyoto, Japan) and a model TNM-1 analyzer (Shimadzu Corp., Kyoto, Japan), respectively. All measurements were conducted in triplicate.

## Method validation

### CFR temperature and pressure profiles

**Fig. 3:**
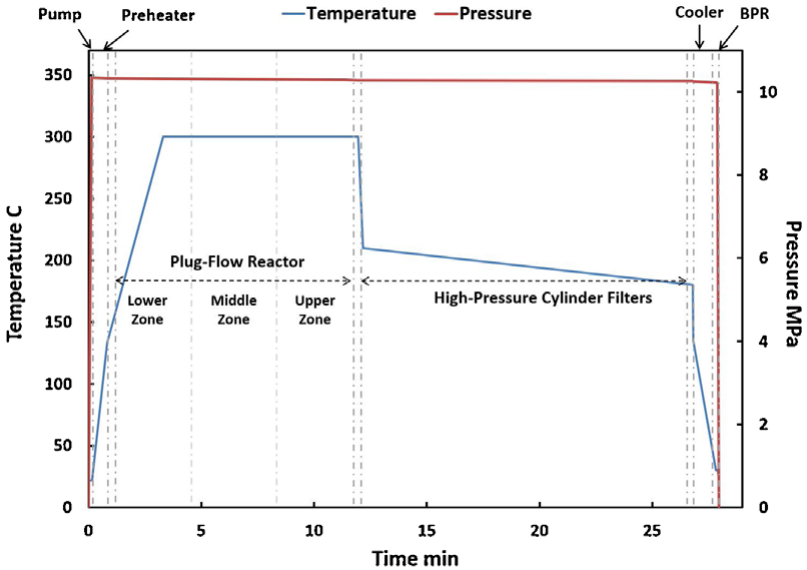
Temperature (blue curve) and pressure (red curve) profiles of water flow in the CFR system under a reaction temperature of 300 °C.

The temperature profile in the CFR system when the reactor temperature was set to 300 °C is shown in Fig. 3. The flow was heated by the preheater from 30 to 133 °C during the 10 s residence time, then to 300 °C in the lower zone of the PFR. After exiting the top of the PFR, the temperature decreased sharply to 230 °C due to the heat loss from the larger diameter (1 cm O.D.) tubing. Solids-containing flow entered the filter vessel at 210 °C and exited (solids-free) after 15 min at 180 °C. The heat exchanger lowered the temperature to 30 °C before the BPR.

The operational temperature profiles of the preheater (133 °C) and three band heaters (342, 350, and 350 °C, respectively) on the PFR, when processing 5 wt.% microalgae at 155 mL/min and 350 °C, indicate that the band heaters and insulation enabled the desired reaction temperature (average heating rate of 150 °C/min and ramping period of < 2 min) (Fig. S4). This heating rate represents a nearly 50× improvement over the batch reaction: 3.2 °C/min heating rate and 112 min ramping period [36,37]. The selected temperature profile was targeted for continuous operation to (1) reduce the likelihood of clogging in the preheater due to premature reactions, (2) keep flow viscosity as low as possible, (3) maintain high efficiency of solids separation within filters vessels, and (4) prevent reactor pressures from exceeding safety thresholds.

**Fig. 4:**
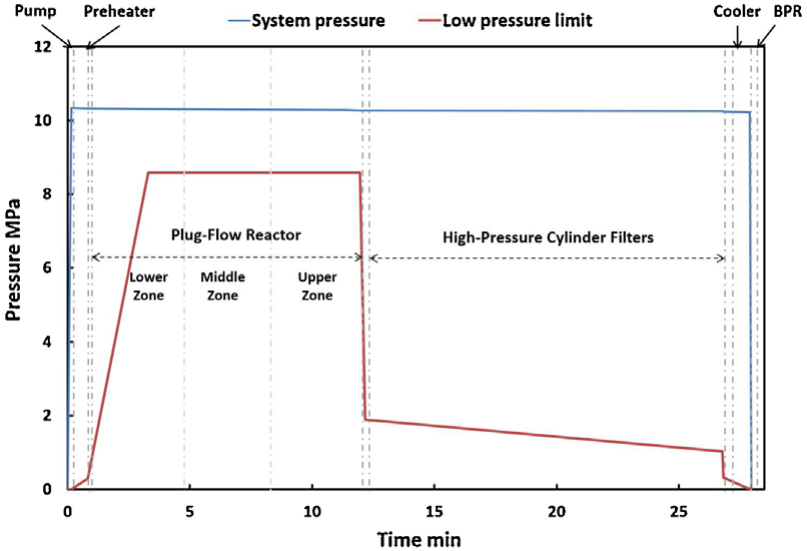
Pressure profile of water (blue curve) compared to the theoretical saturated steam pressure in the CFR system (low pressure limit, red curve) at 300 °C.

**Fig. 5:**
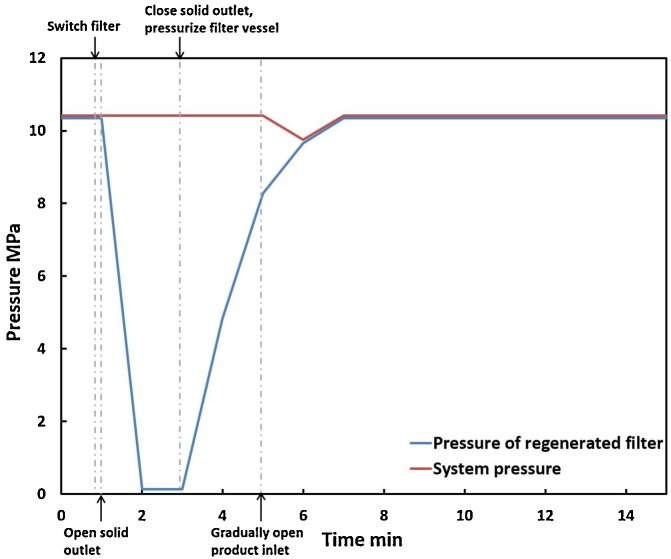
Pressure profiles within the CFR system and a filter vessel during filter cleaning at 300 °C and 10.34 MPa.

The system pressure within the CFR system was highest at the outlet of the pump (10.35 MPa) and decreased until the inlet of the BPR (10.22 MPa), much higher than the saturated steam pressure (the reactor’s “low pressure limit”) at those temperatures (Fig. 4). During filter regeneration, the system pressure dropped slightly to 9.76 MPa, followed by a nearly instantaneous return to normal (Fig. 5), demonstrating the ability for in-situ filter cleaning with little disturbance to system pressure.

### CFR fluid flow profiles and solids loading capability

**Fig. 6:**
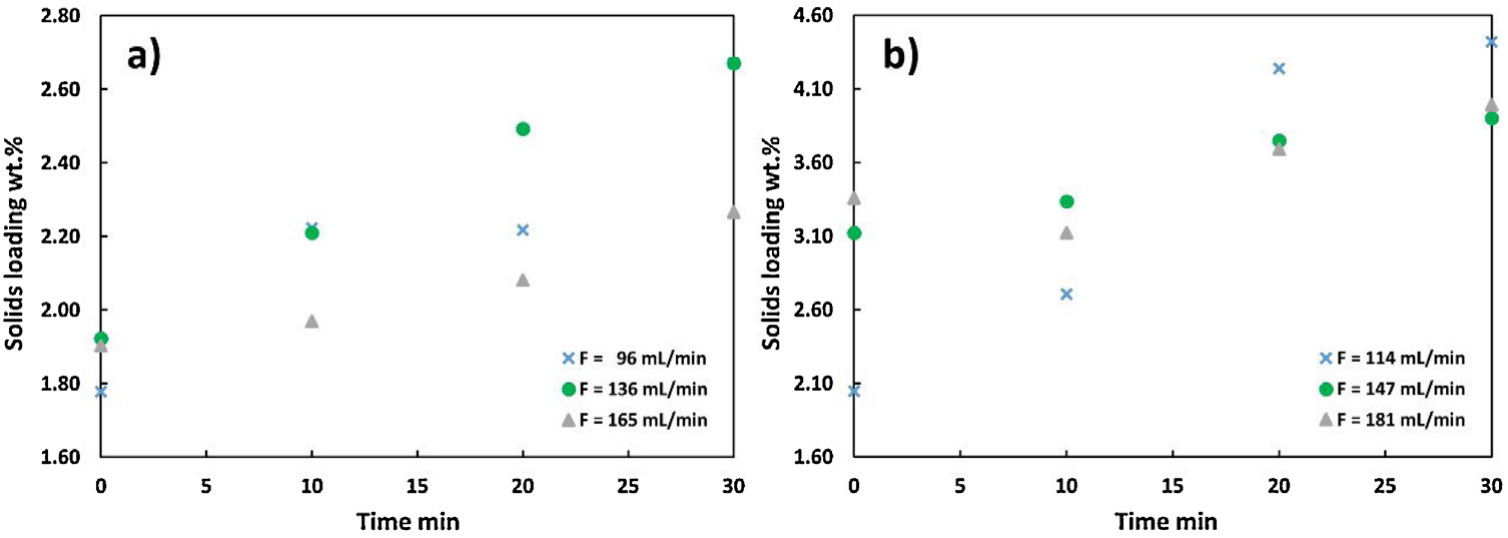
Impacts of back flushing frequency and flow rate on real solids loading of algal slurries with theoretical solids loadings of a) 2.8 wt.% and b) 4.2 wt.%.

Fluid flows in most of the CFR system, except for the flows before the pump and in the main reactor, were characterized by *Re* numbers ranging from 1000 to 9250 (Table S3). Experiments during commissioning showed that there were two areas of particular concern for clogging: before the pump and within the preheater. The higher terminal flow rate before the pump (373 mL/min) was attributed to the larger pipe diameter (20.6 mm I.D.) compared to that after the pump (3.2 mm I.D.). Particle settling in the tubing before the pump, therefore, cannot be avoided by simply increasing the flow rate because the terminal flow rate exceeds the pump’s capability (250 mL/min). To address particle settling prior to the pump, a series of back-flushing schemes was tested (Fig. 6): the real solids loadings of wastewater-grown *Galdieria sulphuraria* microalgae (WWGS) slurries were compared with their theoretical solids loadings (2.8 wt.% and 4.2 wt.%) at the outlet of the pump when different back-flushing frequencies (once every 1, 5, or 30 min) and flow rates (96−181 mL/min) were used. As back-flushing frequency and flow rate decreased, the solids loading did increase. The highest real solids loading measured (4.4 wt.%) exceeded the theoretical value (4.2 wt.%) in the case of the lowest flow rate (114 mL/min) and the least frequent back-flushing (once every 30 min). This indicates, when running solid loadings >4 wt. %, a flow rate <147 mL/min may cause particle settling. One additional observation was that some larger particles were trapped within the cylinder filter, which may have contributed to the real solids loading values being generally lower than the theoretical loadings.

In the preheater, the small diameter (3.2 mm I.D.) tubing makes the preheater coil more susceptible to pressure spikes and clogging at low flow rates and high preheater temperatures, especially in locations where the heat transfer from the band heater is not uniform. Tests run to identify a maximum temperature and minimum flow rate showed that a preheater temperature increase from 133 °C to 165 °C for a 2.8 wt.% algae slurry, and a temperature increase from 133 °C to142 °C for a 4.2 wt.% algae slurry, raised the flow rate at which system pressure began to substantially fluctuate, from 131 to 133 mL/min, and from 135 to 139 mL/min, respectively (Table S4). Therefore, a flow rate of ≥147 mL/min, a pre-pump filter back-flushing frequency of at least every 25−30 min, and a preheater temperature of ≤142 °C were chosen for running an algae slurry of 4.2 wt.% or higher. Reaching continuous operation at more concentrated solids loadings (15−20 wt.%), which have been predicted for economic feasibility, [38] may require additional modifications to tubing diameter, flow rates, and temperatures.

### Residence time distribution in CFR system

**Fig. 7:**
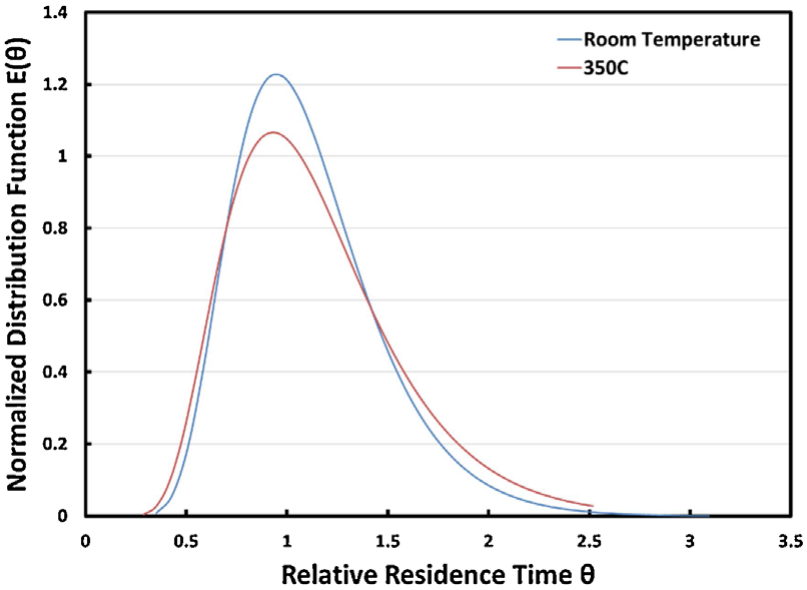
RTD curves of phenol in the CFR system at room temperature and 350 °C. RTD: Residence Time Distribution.

Measured RTD under ambient conditions and under HTL conditions (350 °C and 18 MPa) are shown in Fig. 7 and summarized in Table 1. Little difference in peak broadening at the two temperatures was observed. The maximum peak height (at approximately 21 min for the 350 °C RTD curve) was lower, relating to a phenol concentration of 14.3 mg/L, than that under ambient temperature (23.7 mg/L). Part of the concentration difference was attributed to the slight phenol degradation expected at 350 °C. About 70.8 % of the added phenol exited the CFR system within 80 min at room temperature, while only 56 % of the added phenol exited within the same time at 350 °C. One probable cause of phenol hold up in the system is the difficulty of completely rinsing the inner surface of the filter vessel due to the relatively large volumes. A phenol concentration gradient can lead to diffusion towards the dead zone of the filter vessels; at higher temperatures, diffusion would be faster and therefore, phenol would have a greater chance of diffusing into a dead zone.

**Table 1:** Previous experimental data associated with the RTD of flow.

Ref.	Reactor size	Flow rate mL/min	Time variance	Mean time s	Expected time s	Discrepancy %	*Pe*
Kruse et al. [28]	6 m × 2.1 mm I.D.	27.75	12679	40.6	33.3	22	N.A.
		21.82	58536	54.1	42.4	28	N.A.
		10.62	110	93.6	87	8	N.A.
Mørup et al. [34]	1.2 m × 5.17–25.4 mm I.D.	6–24	311364	33.4 (min)	24.2 (min)	38	28.0
This study, 25 °C	1.83 m × 31.75 mm I.D.	150	86	25.5 (min)	25.7 (min)	0.5	18.4
This study, 350 °C	1.83 m × 31.75 mm I.D.	150	185	31.4 (min)	25.7 (min)	22	13.8

In this study, a very small discrepancy (0.54 %) in the RTD was observed at room temperature; the higher discrepancy at 350 °C (22.2 %) was attributed to the trailing effect observed in other studies. Kruse et al. [28] operated a lab-scale CFR with a lower discrepancy than that of Mørup et al. [34]. Here, the Peclet numbers under room temperature and 350 °C conditions, 18.4 and 13.8, respectively, were lower than that presented in Mørup et al. (28.0), indicating a relatively steady flow throughout this CFR system [34].

### Operation using wastewater microalgae and effects on organic content in HTL aqueous phase

**Fig. 8:**
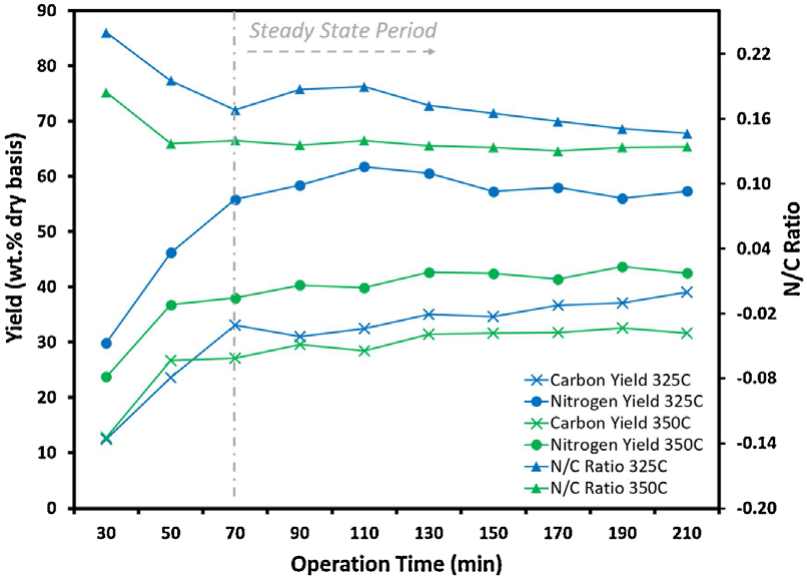
Distributions of C and N yields, and N/C ratio in the HTL aqueous phase from HTL of 5 wt.% WWGS at 325 °C and 350 °C.

Fig. 8 shows that the C and N yields in the HTL aqueous phase reached steady state at approximately 70 min for WWGS. The N/C elemental ratio decreased in the collected liquid products over the course of the reaction, from 0.24 to 0.15 at 325 °C and the shorter residence time, and from 0.18 to 0.13 at 350 °C and the longer residence time. The solids loading of the algae slurry tested here is still lower than the 20 wt.% expected to represent a reasonable trade-off between feedstock dewatering cost and HTL reaction system capital cost [38]. Further modification is needed to improve the capability of the feed supply, reaction, and separation systems to handle algae slurry with higher solids loading. For example, addition of a natural thickener will help mitigate settling of algae particles. Increasing the size of the tubing between the main reactor and the filter system can further reduce the risk of clogging. Installing a heat exchanger will allow recycling of the heat lost from the cooling step to preheat the feedstock stream. Another important issue is the separation of oil and aqueous phase. Without using an organic solvent to obtain bio-crude oil, the viscosity of bio-crude oil is significantly higher, leading to a clogging at the outlet of the oil-aqueous separator [39]. The low lipid content of the wastewater algae feedstock decreases the likelihood of obtaining clear separation of the oil and aqueous phases, resulting in a low oil productivity. Therefore, new strategies need to be developed to address these engineering problems in the future to make larger-scale production economical.

## Declaration of Competing Interest

The authors have no conflicts to declare.
