# Supplementary material for: Modification of a pilot-scale continuous flow reactor for hydrothermal liquefaction of wet biomass
Source: MethodsX. 2019 Nov 19;6:2793–806. doi: 10.1016/j.mex.2019.11.019 (PMC6909004; doi:10.1016/j.mex.2019.11.019)
Supplement: Supplementary file 1 [file mmc1.docx]

# Supplementary material:

# S1. Flow rate estimation

No suitable flowmeter was found for biomass slurry in the CFR, therefore, the flow rate was estimated by collecting samples at the reactor outlet for a set time period. The equation of flow rate, *F*, is shown below:

$F=\frac{V}{t}$ (S1)

where *V* is the volume of sample collected in mL, and *t* is the time in minutes.

The values of Reynolds number (*Re*) were obtained using the equation:

$Re=\frac{du\rho}{\mu}$ (S2)

where *ρ* is fluid density, *u* is the mean fluid velocity (based on flow rate calculation), *μ* is dynamic viscosity, and *d* is the fluid’s hydraulic diameter.

# S2. Heat transfer measurement

An average fluid heating rate was calculated from the flow rate and final temperature using:

$\dot{q}=\frac{T_{f}-T_{i}}{V/F}$ (S3)

where $\dot{q}$ is the heating rate in ºC/min, *T_f_* and *T_i_* are the final temperature and initial temperature in ºC, respectively, *V* is the inner volume of the reactor in mL, and *F* is the volumetric flow rate in mL/min.

The theoretical heat equation for heating wet biomass is:

$\dot{Q}_{req}=\dot{m}C_{p}\Delta T=\rho\dot{V}C_{p}\left( T_{out}-T_{in} \right)$ (S4)

where *ρ* is water density and *C_p_* is the heat capacity of water (obtained from NIST http://webbook.nist.gov/chemistry/fluid/), $\dot{V}$ is volumetric flow rate, *T_out_* and *T_in_* are outlet temperature and inlet temperatures, respectively, of the lower zone in the PFR.

The equation for heat loss to the environment is:

$\dot{Q}_{Loss}=\frac{T_{Heater}-T_{Film}}{\frac{\ln(r_{3}/r_{2})}{2\pi Lk_{c}}+\frac{\ln(r_{4}/r_{3})}{2\pi Lk_{ss}}+\frac{1}{2\pi r_{4}Lh_{Ext}}}$ (S5)

$h_{Ext}=\frac{C\kappa R_{a}^{n}}{L}$ (S6)

$Ra=Pr\times Gr$ (S7)

$Pr=\frac{\mu C_{p}}{\kappa}$ (S8)

$Gr=\frac{gL^{3}T_{Film}\rho^{2}\beta_{P}}{\mu^{2}}$ (S9)

$\beta_{P}=\frac{1}{T_{Film}}=\frac{2}{T_{Heater}+T_{Air}}$ (S10)

where *r_2_*, *r_3_*, and *r_4_* are the outer diameters of reactor column, the column with ceramic layer, and the column with ceramic and outer shell, respectively. *L* is the length of the PFR lower zone. *T_Heater_* and *T_Film_* are the temperatures of the heater surface and the mean temperature between the heater and the surrounding air (assuming 25 °C), respectively; *T_Heater_* depends on the operating temperature. *k_c_* is the thermal conductivity of ceramic layer, 0.0779 W/(m·K), and *k_ss_* is the thermal conductivity of the stainless steel outer shell, 16 W/(m·K). *h_Ext_* is the heat transfer coefficient of the surrounding air. *Ra*, *Pr*, and *Gr* are the Rayleigh, Prandtl, and Grashof numbers, respectively, *g* is gravitational acceleration, and *β_P_* is isobaric compressibility (ideal gas). *μ*, *C_p_*, *κ*, and *ρ* are dynamic viscosity, heat capacity, conduction heat transfer coefficient, and density of the surrounding air, respectively. *C* and *n* are the constants for convection coefficient, 0.59 and 0.25, respectively, since the air flow is laminar (*Ra* < 10^9^).

The equation for calculating total power required for heating biomass is:

$\dot{Q}_{Total}=\dot{Q}_{req}+\dot{Q}_{Loss}$ (S11)

The equation of energy loss is:

$\dot{Q}_{RL}=\dot{Q}_{RT}-\dot{Q}_{Total}$ (S12)

where $\dot{Q}_{RT}$ is the real total power provided by the ceramic band heater.

# S3. Estimation of terminal flow rate and terminal velocity

The terminal velocity of flow in the tubing can be measured according to Newton’s law:

$W=F_{B}+F_{D}$ (S13)

where *W* represents the particle weight, *F_B_* and *F_D_* represent the buoyancy force and drag force, respectively, acting on the particle. Approximating algae particle as a smooth sphere, the specific terms for the three forces are:

$W=\frac{\pi}{6}\rho_{p}gd^{3}$ (S14)

$F_{B}=\frac{\pi}{6}\rho_{w}gd^{3}$ (S15)

$F_{D}=\frac{1}{2}C_{D}\rho_{w}v^{2}\frac{\pi}{4}d^{2}$ (S16)

where *ρ_p_* is particle density, *ρ_w_* is fluid density, *g* is gravitational acceleration, *d* is particle diameter, *C_D_* is drag coefficient (equal to 0.445 for 750 < *Re* < 350,000), and *v* is terminal velocity.

The terminal velocity was estimated as:

$v=\sqrt{\frac{4gd}{3C_{D}}\left( \frac{\rho_{p}-\rho_{w}}{\rho_{w}} \right)}$ (S17)

The terminal flow rate was obtained via multiplying terminal velocity by the cross-sectional area of the tubing.

# S4. Additional Tables and Figures

**Table S1.** Components of the CFR system.

| **Component** | **Company, Location** | **Material** | **Rated Pressure (bar) / Temperature (°C)** |
| --- | --- | --- | --- |
| C-Clamp Mount Agitator | Grovhac, Inc., Brookfield, WI, USA | Stainless Steel (SS) | N.A. |
| Inductor Cone Bottom Tank | Den Hartog Industries, Inc., Hospers, IA, USA | Medium-Density Polyethylene (U.V. Inhibitors) | N.A. / 54 °C |
| Model 4 Basket Strainer (Cylinder Filter) | Rosedale Products, Inc., Ann Arbor, MI, USA | Wetted Parts (316 SS), Viton Cover and Basket Seals (Teflon) | 34.47 bar / 204.44 °C |
| The tube and fittings between the feed tank and the pump | The Home Depot, Inc., Atlanta, GA, USA | Polyvinyl Chloride (PVC) | 33.09 bar / 60 °C |
| Milroyal B Metering Pump (Packed Plunger Liquid End) | Milton Roy, LLC., Warminster, PA, USA | Liquid End and Crosshead (316 SS), Packing/Plunger (Teflon/Ceramic) | 444.71 bar / 60 °C |
| Sentry XPH Pulsation Dampener | Blacoh, Riverside, CA, USA | Body (316 SS), Bellows (PTFE), Bladders (Buna-N, EPDM, Viton) | 345 bar / 104 °C |
| The Tube and Fittings between the Pump and the Preheater | High Pressure Equipment Co., Erie, PA, USA | 316 SS | 689.48 bar / N.A. |
| The Valve and Fittings between the Pump and the Preheater | Swagelok, Solon, OH, USA | 316 SS | 689.48 bar / 232 °C |
| Model T2 Pressure Transducer | Ashcroft, Stratford, CT, USA | Pressure Connection and Sensor Diaphragm (304 SS) | 344.74 bar / 125 °C |
| Preheater Coil and Band Heater | Supercritical Fluid Technologies, Inc., Newark, DE, USA | Stainless steel strap, ceramic insulation | 4136.85 bar / N.A. |
| Plug-Flow Reactor (1.45 L) | High Pressure Equipment Co., Erie, PA, USA | Wetted Surface (304 SS), alloy steel outside (non-wetted) caps, BUNA-N (nitrile) O-Ring Closure | 344.74 bar / 400 °C |
| Band Heaters in Plug-flow Reactor | Industrial Heater Co., Cheshire, CT, USA | Ceramic Band, Flange lock up |  |
| The Tube and Fittings between Plug-Flow Reactor and Blowdown Pots | Parker, Cleveland, OH, USA | Wetted Surface (316 SS) | 537.79-689.74 bar @ 37.78 °C (435.61-558.48 bar @ 260 °C) |
| Customized High-Pressure Filter Housing (1.9 L) | Parr Instrument Company, Moline, IL, USA | T316 SS, Flat Gasket (PTFE) | 200 bar @ 350 °C |
| Customized High-Pressure Filter Element | Rosedale Products, Inc., Ann Arbor, MI, USA | 316 SS | 34.47 bar @ 204.44 °C |
| Differential Pressure Gauge | REOTEMP Instruments, San Diego, CA, USA | Housing (Aluminum), Wetted Parts: Gauge body/Internals (Aluminum/SS), Internal Seals (Buna) | 206.84 bar / 93.33 °C |
| Customized High-Pressure Blowdown Pot | Parr Instrument Company, Moline, IL, USA | T316 SS, O-ring (Viton) | 200 bar @ 250 °C |
| The Tube and Fittings between the High-Pressure Filter Housing and the Cooler | High Pressure Equipment Company, Erie, PA, USA | 316 SS | Tee and Needle Valves (2068.43 bar), Tube (4136.85 bar) |
| TLR4225 Sample Cooler | Sentry Equipment Corp, Oconomowoc, WI, USA | 316 SS | Inner Tube (345 bar @ 538 °C), Shell (31 bar @ 343 °C) |
| The Valve, Tube and Fittings after the Cooler | Swagelok, Solon, OH, USA | 316 SS | 562.61-760 bar @ 37.78 °C |
| Back Pressure Regulator | Equilibar, LLC, Fletcher, NC, USA | Body (316 SS), Diaphragm (PTFE), O-Ring (Viton) | 206.84 bar / 70 °C |
| Product Tank | Supercritical Fluid Technologies, Inc., Newark, DE, USA | Drun (304 SS), Gasket (EPDM) | N.A. |
| Air Filter | Wilkerson Corp., Richland, MI, USA | Body (Zinc), Bowl (Aluminum) | 17.24 bar / 65.56 °C, Airflow (8.50 × 105 mL/min @ 6.89 bar) |
| Rupture Discs for the CFR System | Zook, Chagrin Falls, OH, USA | 316 SS | 407.34 bar @ 22 °C (313.64 bar @ 350 °C) |
| Rupture Discs for the Filter Housing | Fike, Blue Springs, MO, USA | Inconel | 206.84 bar @ 22 °C (191.54 bar @ 350 °C) |
| The Tube and Fittings in the CFR rupture disc system | Parker, Cleveland, OH, USA | 316 SS | 537.79-689.74 bar @ 37.78 °C (435.61-558.48 bar @ 260 °C) |
| High Pressure Gas Booster System | MAXPRO Technologies Inc. Fairview, PA, USA | Pump Body (316L SS), Piston (440 SS), Fittings (316 SS) | 299.92 bar / 98.89 °C |
| The Valve, Tube, and Fittings in the CFR N2 supply system | McMaster-Carr, Elmhurst, IL, USA | Valve (316 SS, Brass, and PTFE), Check Valve (Brass, Buna-N), Tube and Tee (316 SS) | 206.84-282.68 bar / 82.22-648.89 °C |
| Micro-controller X Model PXR4 | Fuji Electric Co., Ltd., Tokyo, Japan | Case (Plastic) | 50 °C |
| Solid State Relay SSR330 | OMEGA Engineering, Inc., Norwalk, CT, USA | Case (Plastic) | N.A. |
| Frequency Inverter Drive SMVector NEMA 4X (IP65) Indoor / Outdoor | Lenze Americas, Uxbridge, MA, USA | Housing (Plastic) | 55 °C |
| High Temperature Thermocouples | OMEGA Engineering, Inc., Norwalk, CT, USA | Sheath (Iron-Constantan 304 SS), Connector (Ceramic) | Ceramic Connector (650 °C), Sheath Probes (1335 °C) |
| Auto Relief Valve | LCM Industries, Inc. Odessa, TX, USA | 316 SS | Set Pressure (200 bar) |
| Pressure Gauges | Stewarts-USA, Houston, TX, USA | 316 SS | 344.74 bar |

**Table S2.** Energy consumption in the lower-zone heater of PFR.

| **Operating condition window in the lower zone of PFR** | **350 °C Flow** |
| --- | --- |
| T_inlet_ (Temperature at the bottom of the lower zone) °C ^a^ | 133 |
| T_outlet_ (Temperature at the top of the lower zone) °C ^a^ | 350 |
| Theoretical power for heating algae W ^b^ | 2150 |
| Theoretical energy loss flow toward atmosphere W ^b^ | 50 |
| Real energy loss flow toward atmosphere W ^b^ | >580 |
| Real total power for heating biomass W | >2780 |

^a^ Obtained from the experimental data. ^b^ Equation details in the supplemental information; physical data of water were obtained from http://webbook.nist.gov/chemistry/fluid/.

**Table S3.** Reynolds number and the terminal flow rate throughout the CFR system under a reaction temperature of 300 °C.

|  | **Feed supply system before pump** | **Between pump and preheater** | **Between preheater and reactor** | **Lower part of reactor** | **Middle part of reactor** |
| --- | --- | --- | --- | --- | --- |
| **I.D. cm** | 2.03 | 0.32 | 0.32 | 3.18 | 3.18 |
| ***Re* ^b^** | 163 | 1046 | 2625 | 727 | 876 |
| ***F* mL/min ^a, c^** | 372.84 | 5.11 | 5.36 | None ^d^ | None ^d^ |
|  | **Upper part of reactor** | **Between reactor and filters** | **Between filters and cooler** | **Cooler** | **After cooler** |
| **I.D. cm** | 3.18 | 0.47 | 0.21 | 0.39 | 0.32 |
| ***Re* ^b^** | 876 | 5547 | 9256 | 2177 | 1044 |
| ***F* mL/min ^a, c^** | None ^d^ | None ^d^ | No particles | No particles | No particles |

^a^ Terminal flow rate. Assume the particle size before the plug-flow reactor is 20 µm, while the physical data of particles after the preheater were unavailable, due to unknown density of HTL solids. ^b^ Physical data of water were obtained from http://webbook.nist.gov/chemistry/fluid/. ^c^ Physical data of algal slurry were obtained from the experimental measurement. ^d^ No valid data could be obtained due to unknown physical properties of reactants and products in HTL reaction.

**Table S4.** The low limit of flow rate under different solids loadings and preheater temperatures.

| **Solids loading (wt.%)** | **Preheater temperature (°C)** | **Low limit of flow rate (mL/min)** |
| --- | --- | --- |
| 2.8 | 133 | 131.41 |
|  | 165 | 133.03 |
| 4.2 | 133 | 135.74 |
|  | 142 | 139.04 |


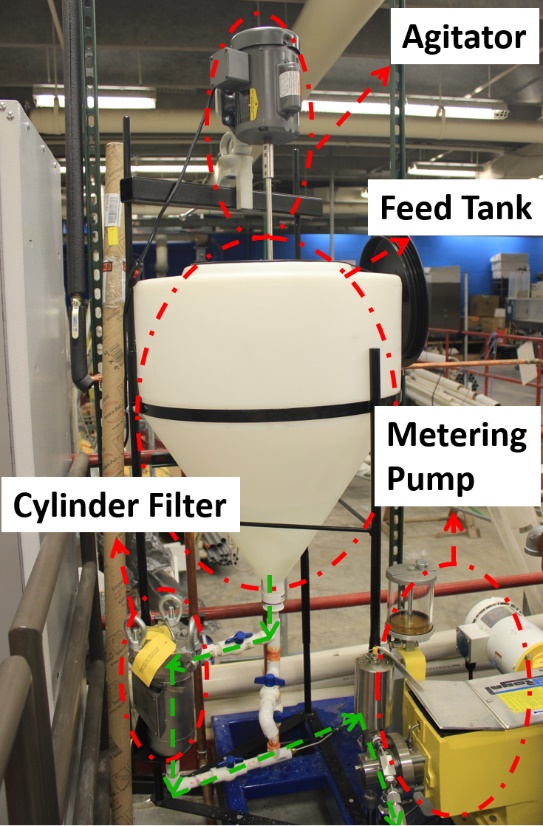


**Figure S1.** Components of the supply system.


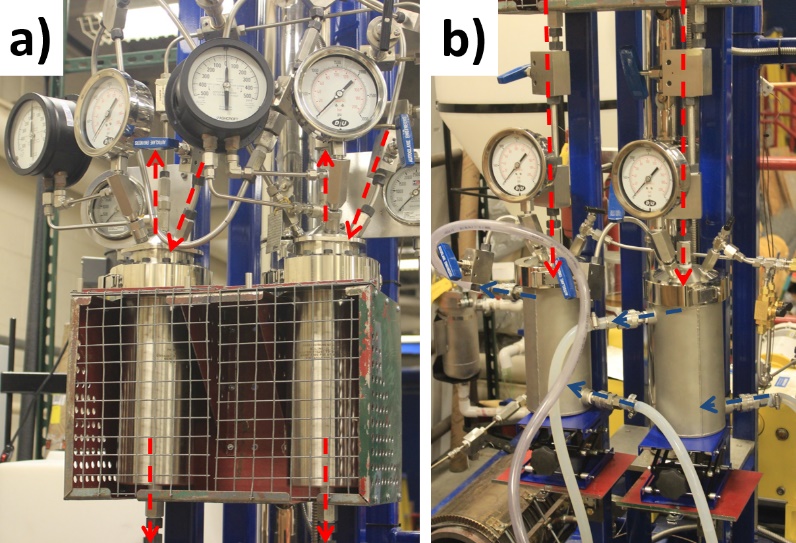


**Figure S2.** Schematic diagrams of (a) the filter vessels and (b) the blowdown pots.


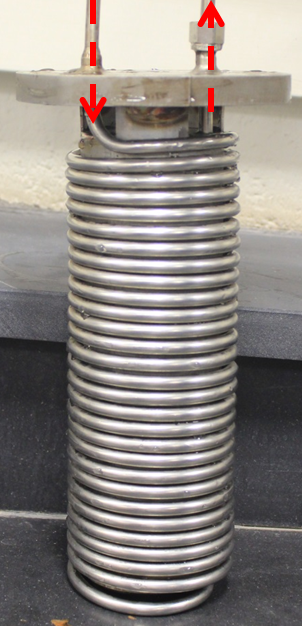


**Figure S3.** Inner structure of the cooler.


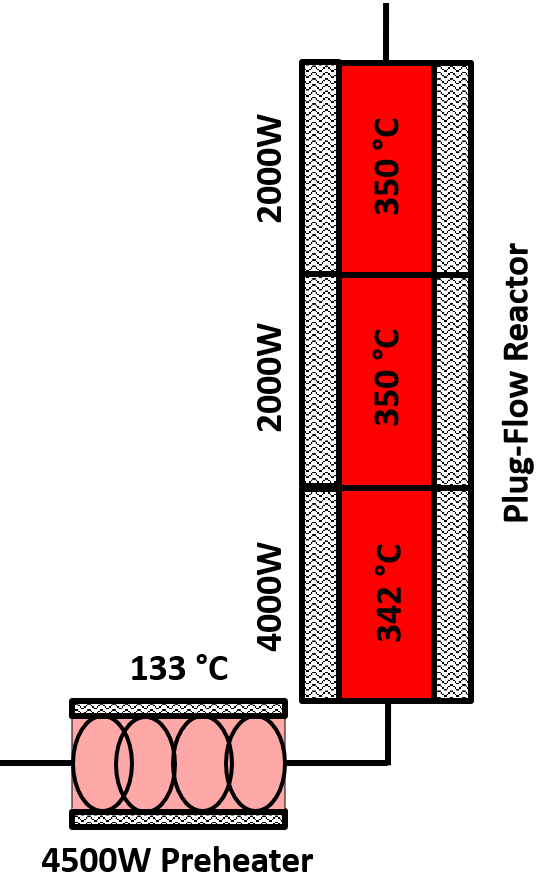


**Figure S4.** Temperature profile of the CFR reacting system were obtained from HTL of wastewater-grown microalgae under a reaction temperature of 350 °C.
